# Supplementary material for: Association between post-stroke depression and functional outcomes: A systematic review
Source: PLoS One. 2024 Aug 22;19(8):e0309158. doi: 10.1371/journal.pone.0309158 (PMC11341015; doi:10.1371/journal.pone.0309158)
Supplement: S1 Table — (DOCX) [file pone.0309158.s001.docx]

**S1 Table. Search strategies.**

| **Database (Search time)** | **Search** | **Search String** |
| --- | --- | --- |
| PubMed and MEDLINE (January 17, 2024) | 1 | (("Stroke"[Mesh] OR "Hemorrhagic Stroke"[Mesh] OR "Ischemic Stroke"[Mesh] OR "Embolic Stroke"[Mesh] OR Cerebrovascular accident[tiab] OR Cerebrovascular disease[tiab] OR Cerebrovascular disorders[tiab] OR "Brain Ischemia"[Mesh] OR "Cerebral Infarction"[Mesh] OR Cerebral ischemia[tiab] OR "Cerebral Hemorrhage"[Mesh] OR "Intracranial Hemorrhage, Hypertensive"[Mesh] OR Post-stroke[tiab])) |
|  | 2 | (("Depression"[Mesh] OR Depressive Symptom*[tiab] OR Emotional Depression[tiab] OR Depressive Symptom*[tiab] OR Unhappiness[tiab] OR Sadness[tiab])) |
|  | 3 | ((Functional Outcome[tiab] OR functional recovery[tiab] OR functional improvement[tiab] OR functional impairment[tiab] OR "functional status"[MeSH] OR functional status"[tiab] OR "physical functional performance"[MeSH] OR physical functional performance[tiab])) OR "Disabled Persons"[Mesh])) OR disability[tiab] OR neurological deficit[tiab])) |
|  | 4 | 1 AND 2 AND 3 (n=114) |
| Web of Science (January 17, 2024) | 1 | (TS=(Stroke OR cerebrovascular accident) OR cerebrovascular disease OR cerebrovascular disorders OR cerebral ischemia OR brain ischemia OR cerebral infarction OR ischemic stroke OR hemorrhagic stroke OR Cerebral Hemorrhage OR intracerebral hemorrhage OR post stroke)) |
|  | 2 | (TS=(Depression OR depressive symptoms* OR Emotional Depression OR Depressive Symptom* OR unhappiness OR sadness)) |
|  | 3 | (TS=(Functional Outcome OR functional recovery OR functional improvement OR functional impairment OR functional status OR physical functional performance OR disability OR neurological deficit OR Disabled Persons)) |
|  | 4 | 1 AND 2 AND 3 **(n=1,581)** |
| CINAHL Plus with Full Text (January 17, 2024) | 1 | AB (Stroke OR cerebrovascular accident OR cerebrovascular disease OR cerebrovascular disorders OR cerebral ischemia OR brain ischemia OR cerebral infarction OR ischemic stroke OR Embolic stroke OR hemorrhagic stroke OR Cerebral Hemorrhage OR intracranial hemorrhage, hypertensive OR post-stroke) |
|  | 2 | AB (Depression OR Depressive Symptom* OR Emotional Depression OR Depressive Symptom* OR Unhappiness OR Sadness) |
|  | 3 | AB (Functional Outcome OR Functional Recovery OR Functional Improvement OR Functional Impairment OR Functional Status OR Physical Functional Performance OR Disability OR Neurological Deficit OR Disabled Persons) |
|  | 4 | 1 AND 2 AND 3 **(n=311)** |
