# Supplementary material for: Association between post-stroke depression and functional outcomes: A systematic review
Source: PLoS One. 2024 Aug 22;19(8):e0309158. doi: 10.1371/journal.pone.0309158 (PMC11341015; doi:10.1371/journal.pone.0309158)
Supplement: S2 Table — (DOCX) [file pone.0309158.s002.docx]

**S2 Table. A summary of the included studies.**

| **No.** | **Authors, Year, Country** | **Study Aim** | **Study design** | **N of participants** | **Population** | **Age (years)** | **Measures** | **Measurement Time after stroke** | **Data analysis method** | **Results** |
| --- | --- | --- | --- | --- | --- | --- | --- | --- | --- | --- |
| 1. | Ayerbe et al., 2015  (UK) | To identify explanatory factors for the association between depression at 3 months after stroke and physical disability at 3 years. | Prospective cohort study | N = 1,307  stroke patients aged > 64 who registered in the South London Stroke Register (SLSR)  between the  1 January 1998 and 30 June 2012, up until the 30 June 2013. | - The study included all first stroke patients with an unspecified stroke. | >64 | (1) Depression  - HADS-D (HADS-D depression subscale >7 was categorized as depression)  (2) Functional outcome  - Barthel Index (BI) (0-14 = severe disability; 15-19 = moderate disability; 20 = independent) | (D): 3m, 3y  (F): 3m, 3y | Multinomial regression | - The association between depression at 3 months and severe physical disability was significant when adjusted for age, gender, ethnicity, stroke severity and past medical history of DM, HT, IHD, HF or AF (RR 4.01; 95% CI 2.43-6.62; p<0.001). However, the association was not significant after adjusted for physical disability, cognitive impairment, smoking habit, use of SSRI, subjective perception, or social support at 3 months.  - The association between depression and moderate physical disability was significant (RR 1.89; 95% CI 1.25-2.86; p=0.003).) |
| 2. | Blomgren et al., 2019  (Sweden) | To explore the impact of cognitive dysfunction, emotional problems, and fatigue on long  term  performance of instrumental activities of daily living in young and middle-aged stroke  survivors. | Cross-sectional study | N = 296  Stroke survivors who admitted at the stroke unit of Sahlgrenska University hospital in Gothenburg, Sweden from  1998 to 2003. | - Acute ischemic stroke (first-ever or recurrent acute ischemic stroke) before the age of 70 years. | 18-69 (median age of 64) | (1) Depression  - HADS-D  (2) Functional outcome  (2) Instrumental Activities of Daily Living  - Frenchay Activity Index (FAI) | (D, F): 7y | Logistic regression | - The association between depression and worse outcome in FAI work/leisure was significant with OR 1.10 (95% CI 1.02-1.19; p=0.016) when adjusting for cognitive function (BNIS), gender, living alone, and severity of stroke (NIHSS) for all stroke survivors at their 7 years.  - There was no significant association between depression and FAI domestic chores after 7 years of stroke. |
| 3. | El Husseini et al., 2017 (USA) | To evaluate the independent association between the change in depression rating and the change in mRS. | Multicenter prospective cohort study | N = 1,444  Stroke patients were recruited from hospitals participating in the Get with The  Guidelines (GWTG)–Stroke program from July 2006 to  July 2008. | - Acute ischemic stroke | Median 64 (Q1-Q3 = 55-74) | (1) Depression  - PHQ-8 (>=10 identified as depression)  (2) Functional outcome  - mRS | (D): 3m, 12m  (F): 3m, 12m | Logistic regression | - Patients with resolving depression (from 3 months to 12 months) were less likely to have a worsening functional outcome compared with no depression (OR 0.49; 95% CI 0.29-0.83).  - Compared with no depression, persistent depression and incident depression were not significantly associated with worsening mRS.  - Depression status from 3 to 12 months was associated with change in functional outcome (mRS)(p=0.023).  - Incident depression vs never depressed (OR1.48, 95% CI 0.95-2.30, p-0.010) |
| 4. | Ezema et al., 2019  (Nigeria) | To study the influence  of post-stroke depression on functional independence in activities of  daily living. | Cross-sectional study | N = 66  Stroke survivors who were medically and mentally stable attending outpatient physiotherapy clinic were recruited. | - Unspecified (stroke survivors attending OPD physiotherapy) | Mean 57.52±11.35; range 30-69 | (1) Depression  - HDRS  (2) Functional outcome  - BI (ADL) | (D, F): 1.75y | Chi-square test, unpaired t-test | - PSD was associated with poor level of functional independence in ADL (BI group) (Chi-square test, p<0.001).  - PSD was associated with functional outcome (BI) scores (t-test, p=0.003).  - There was no association between depression and functional independence in ADL after controlling for age and duration of stroke. |
| 5. | Ghaffari et al., 2021 (Iran) | To determine factors predicting instrumental activities of daily living performance in patients with  stroke. | Cross-sectional study | N = 90 (82 for data analysis)  Patients with first stroke were recruited from 5 occupational therapy clinics from March 2019 to December 2019. | - Unspecified (first-ever stroke included, excluded TIA)  🡪 Ischemic and hemorrhagic | - 30-80 | (1) Depression  - BDI  (2) Functional outcome  - Lawton IADL  - BI | N/A | t-test,  ANOVA,  Pearson correlation,  Multiple linear regression | - Depression was associated with IADL (r= -0.758; p<0.001).  - Depression (p=0.045) was identified as one of significant predictors of IADL performance when controlled for age, TMT, and BADL performance (R2=74.13%). |
| 6. | Kang et al., 2018  (Korea) | To estimate the effects of acute phase PSD on functional outcomes during the acute and chronic phases of recover. | Prospective cohort study | N = 423  Stroke patients who were hospitalized at Chonnam National  University Hospital. | - Acute ischemic stroke | - No PSD 63.8±10.1  - PSD 66.6±9.7 | (1) Depression  - Mini International Neuropsychiatric Interview  (2) Functional outcome  - BI  - mRS | (D): 2w  (F): 1y | Repeated measures ANOVA, Multiple linear regression,  ANCOVA | - PSD in the acute phase (2 weeks) was significantly associated with NIHSS during the chronic phase (1 year) after adjusting for NIHSS in the acute phase (MLR, beta 0.152; p=0.005).  - PSD in the acute phase (2 weeks) was significantly associated with BI during the chronic phase (1 year) after adjusting for BI in the acute phase (MLR, beta -0.162; p=0.003).  - PSD in the acute phase (2 weeks) was significantly associated with NIHSS during acute phase (2 weeks) and chronic phase (1 year) after adjusting for age, previous depression and stroke, stroke hemisphere, and stroke location (ANCOVA, ps<0.001).  - PSD in the acute phase (2 weeks) was significantly associated with BI during acute phase (2 weeks) and chronic phase (1 year) after adjusting for age, previous depression and stroke, stroke hemisphere, and stroke location (ANCOVA, p<0.001). |
| 7. | Kapoor et al., 2019  (Canada) | To evaluate the ability of this validated brief depression, obstructive sleep apnea (OSA), and cognitive  impairment (DOC) screening tool to reintegration into community roles  and IADL 2 to 3 years after stroke. | Prospective cohort study | N = 124  Stroke patients who were recruited into to DOC Feasibility Study between April 2012 and April  2014. | - Ischemic stroke  - Hemorrhagic stroke | Mean 66.3±15.7 | (1) Depression  - PHQ-2  - CES-D  (2) Functional outcome  - FAI  - RNRI | (D): <6m  (F): 2-3y | Linear regression analysis,  Logistic regression,  Pearson’s correlation | - More depressive (PHQ-2 scores) (beta = -2.41; p=0.023) was one of significant predictors of poor instrumental activity together with age, stroke severity, and cognitive symptoms.  - Depression risk (PHQ-2 scores) was the only significant predictor of participation on the RNLI (OR 0.46; p=0.028) when controlled variables of age, stroke severity, and cognition.  - CES-D score significantly correlated with RNLI (r=0.523; p<0.001), but not FAI (r=-0.206, p=0.190). |
| 8. | Karaahmet et al., 2017  (Turkey) | To examine the  risk factors influencing the development of post-stroke depression and to determine the effect of depression on the patients’ functional improvement after rehabilitation. | Prospective cohort study | N = 93  Stroke patients attending the Physical Medicine and  Rehabilitation. | - Ischemic stroke  - Hemorrhagic stroke | Mean 58.7±13.8 | (1) Depression  - BDI  (2) Functional outcome  - FIM | (D, F): <6m | Mann-Whitney U test | - FIM changes from admission to follow-up (1-month) were higher in patients with no depression compared to those who had depression after stroke (p<0.05). |
| 9. | Kim et al., 2022  (Korea) | To evaluate the relationship between cognitive function and functional outcome after controlling for depression. | Longitudinal cohort study | N = 423  Patients who were hospitalized due to ischemic stroke from  January 2017 to April 2019 and underwent a cognitive function test in the stroke registry at Kangwon National University Hospital,  Republic of Korea. | - Ischemic stroke | Mean 73.5±10.1 | (1) Depression  - GDS treated as a controlled factor  (2) Functional outcome  - mRS (changes in functional outcome from discharge to 12 months) | (D): discharge, 3m, 12m  (F): discharge, 3m, 12m | A linear mixed-effects model | - The main analysis is exploring effect of cognitive function on change in functional outcome (mRS) and depression was treated as controlled factor.  - GDS had significant effect (p=0.032) on change in mRS in a linear mixed-effect model containing MMSE, time, MMSExtime, age, sex, initial NIHSS, HT, DM, DLP, AF, and IV thrombolysis for all stroke participants.  - However, when considered mRS change by severity of stroke, there was no significant effect of GDS on mRS changes found in minor, moderate, and severe stroke.  - Total case in final analysis was 398 cases. The authors did not explain about lost cases.  - not sure which time points of measuring GDS they used for a linear mixed-effects model. |
| 10. | Li et al., 2019  (China) | To investigate the clinical and neuroimaging risk factors for development of post stroke anxiety  (PSA) and examine the effects of PSA on activities of daily living (ADL) and quality of life (QOL) in Chinese patients with ischemic stroke. | Prospective cohort study | N = 219  Patients with first-ever or recurrent acute ischemic stroke  admitted to the Department of Neurology, Dongguan People  Hospital, between July 2013 and June 2014. | - Acute ischemic stroke (first-ever or recurrent) | 40 – 80 | (1) Depression  - HDRS (Hamilton Depression Rating Scale)  (2) Functional outcome  - Lawton ADL scale | (D): 5-14d  (F): 3m | Multiple linear regression | - HDRS in the acute stage of stroke was significantly associated with 3-month ADL when adjusting for age, sex, NIHSS, and HARS in the acute stage.  - Patients with more depressive symptoms were more likely to have a poorer performance in ADL (adjusted beta=0.281; p<0.001). |
| 11. | Lopatkiewicz et al., 2021  (Poland) | To determine  whether outcomes vary among patients with different trajectories of post-stroke depressive symptoms. | Prospective cohort study | N = 335  Stroke patients who registered to the PROPOLIS study (PRospective Observational POLIsh  Study on post-stroke delirium) between  May 2014 and March 2016. | - Acute ischemic stroke  - TIA | Median 68 | (1) Depression  - PHQ-9 (Patient  Health Questionnaire-9)  (2) Functional outcome  - mRS (modified Rankin Scale) (3-6 defined as unfavorable functional outcome) | (D): 8d, 3m  (F): 3m, 12m | Logistic regression | - Later depressive symptoms (at 3 months) had an increased risk of poor 3-month functional outcome (aOR 2.59; 95% CI 1.64-4.07) when controlled age, NIHSS score, pre-stroke cognitive decline, NPI score (Neuropsychiatric Inventory), and delirium.  - Later depressive symptoms (at 3 months) had an increased risk of poor 12-month functional outcome (aOR 3.97; 95% CI 2.32-6.76) when controlled age, NIHSS score, HT, AF, pre-stroke cognitive decline, NPI score, and delirium. |
| 12. | Lv et al., 2021  (China) | To explore the rate of post-stroke disability and its associated factors in the third year following discharge from inpatient rehabilitation in Northeast China. | Prospective cohort study | N = 522 Stroke patients were hospitalized in the department of rehabilitation from 4 hospitals in Daqing between April 2015 and December 2015. | - Ischemic stroke  - Intracerebral hemorrhage  - Mixed | ≥18 | (1) Depression  - Self-rating depression scale (SDS)  (2) Functional outcome  - Modified BI (≤95 indicated disability) | (D, F): 3y | t-test,  Logistic regression | A total of 448 participants were used for the logistic regression.  - In the 3^rd^ year of follow-up, patients with disability had higher depression scores than those without disability (43.98 (10.30) vs. 35.89 (8.97); p<0.001)  - Multiple logistic regression analysis showed that depression was a significant factor in disability after 3 years of stroke (OR 1.033; 95% CI 1.007-1.061) after adjusting for age, stroke severity, cognition, and social support. |
| 13. | Matsuzaki et al., 2015  (Japan) | To investigate the relationship between the clinical condition of post-stroke depression (PSD) and the physical recovery of stroke patients in a rehabilitation hospital. | Prospective cohort study | N = 153 stroke patients who were admitted to Kumamoto Takumadai Rehabilitation Hospital between July 2011 and June 2013. | - Unspecified (excluded SAH, TIA) | Mean 71.9±13.8 | (1) Depression  - Self-rating depression scale (SDS)-subjective  - SDS>=40 indicated a depressed group  - the Montgomery-Asberg Depression Rating Scale (MADRS-J)-objective  (2) Functional outcome  - FIM (Functional Independence Measurement) recovery (FIM score on discharge – FIM score on admission) | (D): 10d  (D): 4-6 w  (F): 4-6 w | 2-Way ANOVA | - A total of 117 participants were used for analysis.  - The subjective depression scale (SDS) had no significant association with the physical recovery of stroke survivors from admission to discharge (p=0.095) adjusted for gender, age, LOS, FIM score on admission, MMSE, and apathy.  - Objective depression scores (MADRS-J) showed a significant impact on the recovery of physical and cognitive function after controlling confounding factors. |
| 14. | Mohammed et al., 2023  (Ethiopia) | To examine the magnitude and predictors of post-stroke limitation in basic and instrumental activities of daily living. | Cross-sectional study | N = 150  Stroke survivors with a stroke duration of more than 6 months from Tikur Anbessa Specialized  Hospital (TASH) between April and October 2022. | - Ischemic stroke  - Hemorrhagic stroke | - Age >18  - Mean 53±14.9 | (1) Depression  - PHQ-9 (Patient Health Questionnaire-9) (>=10 indicated depression)  (2) Functional outcome  - Basic activities of daily living (BADL) using the Barthel Index (BI)  - Instrumental activities of daily living using  Frenchay Activities Index (FAI) | (D): >6m  (F): >6m | Logistic regression | - Depression was a predictor of severe limitation in IADL (aOR 5.1; 95% CI 1.1-23.2) after controlling for age, stroke duration, history of substance use, comorbid, aphasia, cognitive impairment, and initial NIHSS. |
| 15. | Nakamori et al., 2020  (Japan) | To investigate the relationship between PHQ-9 score and functional outcomes in stroke patients in a convalescent rehabilitation ward. | Retrospective cohort study | N = 258 stroke patients who were admitted to the convalescent rehabilitation ward at the Suiseikai Kajikawa  Hospital, Hiroshima, Japan during the period from 1 January 2017  to 30 September 2019. | - Ischemic stroke  - Hemorrhagic stroke  - Others | Mean 72.0±12.9 | (1) Depression  - PHQ-9 (Patient Health Questionnaire-9) (>5 depression)  (2) Functional outcome  - FIM (Functional Independence Measure) gain score (last FIM score – first FIM score) | (D, F): <4 months (Mean of staying rehabilitation wards 82d) | Multiple linear regression | - A total of 215 participants was used for analysis.  - PHQ-9 score on admission to the rehabilitation ward was a significant factor influencing the FIM gain score (beta= -0.745; p=0.009) adjusted for age, sex, solitude, comorbid diseases, NIHSS, stroke subtypes, location of lesion, MMSE, and FIM on admission.  - PHQ-9 score on admission was also a significant factor in predicting inability to discharge (OR 1.24; 95% CI 1.12-1.39). |
| 16. | Paolucci et al., 2019  (Italy) | To assess the specific influence of post-stroke depression (PSD) and antidepressant treatment on both basal functional status and rehabilitation outcomes. | Case-control study | N = 560 (280/280)  Patients with sequelae of a stroke event were admitted to the rehabilitation unit between January 2004 and December 2018. | - Ischemic stroke | - Non-PSD: Mean 70.44±9.97  - PSD: Mean 70.52±10.03 | (1) Depression  - BDI (>=10 depression)  - DSM-V  (2) Functional outcome  - modified BI  - Rivermead Mobility Index (RMI) | (D): during hospital stay  (F): Non-PSD (Mean 82.27d) (F):  PSD (Mean 88.62d) | Wilcoxon Signed Ranks Test | - This study focused on antidepressant drugs (AD) response.  - BI and BDI scores were treated as independent variables.  - However, PSD patients who responded to AD treatment had higher BI score at discharge than those non-responded to AD treatment (59.40±30.56 vs. 44.50±28.50; p=0.009) |
| 17. | Schöttke et al., 2020  (Germany) | To investigate the relationship between post stroke depression (PSD), functional impairments (FI),  and social support of stroke patients in a 3-year prospective design. | Prospective cohort study | N = 174  Stroke patients from three German rehabilitation clinics. | - Acute ischemic stroke  - Intracerebral hemorrhage | Mean 67.51±11.63 | (1) Depression  - DSM-IV (Diagnostic and Statistical Manual of  Mental Disorders)  - CDS (Cornell Scale for Depression)  (2) Functional outcome  - BI (Barthel-Index) | (D): mean 6.5w (T1), 3y (T2)  (F): mean 6.5w (T1), 3y (T2) | OLS regression model | - PSD at acute phase of stroke significantly increased functional impairment after 3 years of stroke (beta -16.47; p=0.001) when controlling for sex, age, and functional impairment on acute stroke. |
| 18. | Sharma et al., 2021  (India) | To observe the effect of post-stroke depression on  Functional outcomes during inpatient rehabilitation. | Prospective cohort study | N = 30  Patients who experienced their first stroke with <1 year duration and attended the neurorehabilitation unit of a tertiary hospital between October 2019 and April 2020. | - Ischemic stroke  - Hemorrhagic stroke | ≥18 (Median 58) | (1) Depression  - HADS-D (Hospital Anxiety and Depression Scale)  (>7 classified depression)  - HDRS  (2) Functional outcome  - BI (Barthel-Index)  - mRS (modified Rankin Scale)  - SSS (Scandinavian Stroke Scale) | (D): <1y  (F): <1y | Mann-Whitney U test,  Spearman’s rank correlation | - Participants with and without depression showed improvement in all the functional outcome measures (BI, SSS, mRS) at the time of discharge as compared with admission scores.  - There was no significant difference in the functional outcomes between stroke patients with depression and those without depression with inpatient rehabilitation program (discharge - admission) (no confounding controlled).  - Age of patients with depression (median 58) and without depression group (median 37) were significantly different (p=0.01).  - Subgroup analysis for patients with depression (n=17) found a significant association between depression score differences and BI differences (discharge – admission), r=0.62, p<0.001 for HADS-D and BI, and r=0.73, p<0.001 for HDRS and BI). |
| 19. | Wang et al., 2018  (China) | To investigate  The association between post-stroke depression (PSD), aphasia, and physical independence in stroke patients in China at 3-month follow-up. | Prospective cohort study | N = 270 patients who had an acute stroke within 14 days from the Stroke Centre of Beijing Tiantan  Hospital between April 2014 and October 2015. | - Acute ischemic stroke | ≥18 | (1) Depression  - SADQ (Stroke Aphasia Depression Questionnaire)  (2) Functional outcome  - mRS (modified Rankin Scale) (>2 indicating physical dependence) | (D): 3m  (F): 3m | Logistic regression | A total of 248 participants were used for analysis.  - Depression at baseline (in hospital) was not associated with physical dependence at 3 months.  - More severe PSD at 3 months was associated with physical dependence at 3 months (OR 1.05; 95% CI 1.01-1.10) when controlling for sex, marital status, stroke type, aphasia at baseline, pulmonary infection at baseline, DVT at baseline, and stroke severity on admission. |
| 20. | Yang et al., 2016  (China) | To conduct a nationwide survey in China to investigate the 5-year prevalence of post-stroke disability and its correlation factors | Prospective cohort study | N = 2,324 acute stroke patients from 56 hospitals who registered in the Prospective  Cohort Study on the Incidence and Outcome of Patients with Post-stroke Depression in China  (PRIOD). | - Ischemic stroke | ≥18 | (1) Depression  - DSM-IV  - HRSD  (2) Functional outcome  - mRS (>=2 indicating physical dependence) | (D): 3m  (F): 5y | Logistic regression | A total of 893 participants were used for analysis.  - Depression at 3 months was associated with post-stroke disability at 5 years (aOR 1.8; 95% CI 1.1-2.9) when controlling for age, sex, education, DM, cardiac disease, smoking, alcohol drinking, stroke history, NIHSS score at admission, cognitive impairment at 3 months, and stroke recurrence within 5 years. |
| 21. | Zeng et al., 2021  (China) | To determine the relationship between early-onset post-stroke depression (PSD) (1 month after stroke) and functional outcomes 5 years after baseline enrollment. | Prospective cohort study | N = 436 stroke patients treated in  the stroke unit of the First Affiliated Hospital of Wenzhou  Medical University from October 2013 to February 2015. | - Acute ischemic stroke | 18-80 | (1) Depression  - HAM-D (Hamilton Depression Rating Scale) (≥7 indicating depression)  (2) Functional outcome  - BI (Barthel-Index)  - mRS (modified Rankin Scale) (>2 indicating unfavorable outcome) | (D): 1m  (F): 5y | Logistic regression | A total of 363 participants were used for analysis.  - The OR for unfavorable outcome at 5 years in the early-onset PSD group was 2.279 times of non-PSD group (95% CI 1.179-4.421) after adjusting for potential risk factors.  - In the early-onset PSD group, early-onset depression severity (HAMD scores) was associated with 5-year unfavorable outcome rates (OR 1.168; 95% CI 1.015-1.345) after adjusting for potential risk factors. |
|  | d, days; w, weeks; m, months, y, years; D, depression assessment; F, functional outcome assessment  aOR = Adjusted odds ratio  DM = Diabetes, HT = Hypertension, IHD = Ischemic Heart Disease, HF = Heart failure, AF = Atrial Fibrillation, DVT = Deep Vein Thrombosis  SSRI = Selective serotonin reuptake inhibitors  BNIS = Barrow Neurological Institute Screen  NIHSS = National Institute of Health Stroke Scale | | | | | | | | | |

**References of included studies**

Ayerbe, L., Ayis, S. A., Crichton, S., Rudd, A. G., & Wolfe, C. D. A. (2015). Explanatory factors for the association between depression and long-term physical disability after stroke. Age and Ageing, 44(6), 1054-1058. https://doi.org/10.1093/ageing/afv132

Blomgren, C., Samuelssonm, H., Blomstrand, C., Jern, C., Jood, K., & Claesson, L. (2019). Long-term performance of instrumental activities of daily living in young and middle-aged stroke survivors-Impact of cognitive dysfunction, emotional problems and fatigue. PLoS One, 14(5), Article e0216822. https://doi.org/10.1371/journal.pone.0216822

El Husseini, N., Goldstein, L. B., Peterson, E. D., Zhao, X., Olson, D. M., Jr.Williams, J. W., Bushnell, C., Laskowitz, D. T., & Williams, J. W., Jr. (2017). Depression status is associated with functional decline over 1-year following acute stroke. Journal of Stroke & Cerebrovascular Diseases, 26(7), 1393-1399. https://doi.org/10.1016/j.jstrokecerebrovasdis.2017.03.026

Ezema, C. I., Akusoba, P. C., Nweke, M. C., Uchewoke, C. U., Agono, J., & Usoro, G. (2019). Influence of post-stroke depression on functional independence in activities of daily living. Ethiopian Journal of Health Sciences, 29(1), 841-846. https://doi.org/10.4314/ejhs.v29i1.5

Ghaffari, A., Rostami, H. R., & Akbarfahimi, M. (2021). Predictors of instrumental activities of daily living performance in patients with stroke. Occupational Therapy International, 2021, Article 6675680. https://doi.org/10.1155/2021/6675680

Kang, H. J., Bae, K. Y., Kim, S. W., Lee, E. H., Kim, J. T., Park, M. S., Cho, K. H., & Kim, J. M. (2018). Impact of acute phase depression on functional outcomes in stroke patients over 1 year. Psychiatry Research, 267, 228-231. https://doi.org/10.1016/j.psychres.2018.06.026

Kapoor, A., Lanctot, K. L., Bayley, M., Herrmann, N., Murray, B. J., & Swartz, R. H. (2019). Screening for post-stroke depression and cognitive impairment at baseline predicts long-term patient-centered outcomes after stroke. Journal of Geriatric Psychiatry and Neurology, 32(1), 40-48. https://doi.org/10.1177/0891988718819859

Karaahmet, O. Z., Gurcay, E., Avluk, O. C., Umay, E. K., Gundogdu, I., Ecerkale, O., & Cakci, A. (2017). Poststroke depression: risk factors and potential effects on functional recovery. International Journal of Rehabilitation Research, 40(1), 71-75. https://doi.org/10.1097/mrr.0000000000000210

Kim, Y., Chung, J. K. Y., Song, J. Y., Jang, H., Jang, J. W., & Kim, S. (2022). Association between baseline cognitive function and longitudinal functional outcome change after ischemic stroke. Dementia and Geriatric Cognitive Disorders, 51(2), 168-174. https://doi.org/10.1159/000523981

Li, W., Xiao, W. M., Chen, Y. K., Qu, J. F., Liu, Y. L., Fang, X. W., Wang, H. Y., & Luo, G. P. (2019). Anxiety in patients with acute ischemic stroke: risk factors and effects on functional status. Frontiers in Psychiatry, 10, Article 257. https://doi.org/10.3389/fpsyt.2019.00257

Lopatkiewicz, A. M., Pera, J., Slowik, A., & Dziedzic, T. (2021). Association of early and later depressive symptoms with functional outcome after ischemic stroke. J Neural Transm (Vienna), 128(5), 679-686. https://doi.org/10.1007/s00702-021-02328-w

Lv, Y. M., Sun, Q. X., Li, J., Zhang, W. Y., He, Y. D., & Zhou, Y. Q. (2021). Disability status and its influencing factors among stroke patients in Northeast China: A 3-year follow-up study. Neuropsychiatric Disease and Treatment, 17, 2567-2573. https://doi.org/10.2147/ndt.S320785

Matsuzaki, S., Hashimoto, M., Yuki, S., Koyama, A., Hirata, Y., & Ikeda, M. (2015). The relationship between post-stroke depression and physical recovery. Journal of Affective Disorders, 176, 56-60. https://doi.org/10.1016/j.jad.2015.01.020

Mohammed, S., Haidar, J., Ayele, B. A., & Yifru, Y. M. (2023). Post-stroke limitations in daily activities: experience from a tertiary care hospital in Ethiopia. BMC Neurology, 23(1), Article 364. https://doi.org/10.1186/s12883-023-03419-9

Nakamori, M., Imamura, E., Tachiyama, K., Kamimura, T., Hayashi, Y., Matsushima, H., Okamoto, H., Mizoue, T., & Wakabayashi, S. (2020). Patient Health Questionnaire-9 predicts the functional outcome of stroke patients in convalescent rehabilitation ward. Brain and Behavior, 10(12), Article e01856. https://doi.org/10.1002/brb3.1856

Paolucci, S., Iosa, M., Coiro, P., Venturiero, V., Savo, A., De Angelis, D., & Morone, G. (2019). Post-stroke depression increases disability more than 15% in ischemic stroke survivors: A case-control study. Frontiers in Neurology, 10, Article 926. https://doi.org/10.3389/fneur.2019.00926

Schöttke, H., Gerke, L., Düsing, R., & Möllmann, A. (2020). Post-stroke depression and functional impairments - A 3-year prospective study. Comprehensive Psychiatry, 99, Article 152171. https://doi.org/10.1016/j.comppsych.2020.152171

Sharma, G. S., Gupta, A., Khanna, M., & Prakash, N. B. (2021). Post-stroke depression and its effect on functional outcomes during inpatient rehabilitation. Journal of Neurosciences in Rural Practice, 12(03), 543-549. https://doi.org/10.1055/s-0041-1731958

Wang, S., Wang, C. X., Zhang, N., Xiang, Y. T., Yang, Y., Shi, Y. Z., Deng, Y. M., Zhu, M. F., Liu, F., Yu, P., Ungvari, G. S., & Ng, C. H. (2018). The association between post-stroke depression, aphasia, and physical independence in stroke patients at 3-month follow-up. Frontiers in Psychiatry, 9, Article 374. https://doi.org/10.3389/fpsyt.2018.00374

Yang, Y., Shi, Y. Z., Zhang, N., Wang, S., Ungvari, G. S., Ng, C. H., Wang, Y. L., Zhao, X. Q., Wang, Y. J., Wang, C. X., & Xiang, Y. T. (2016). The disability rate of 5-year post-stroke and its correlation factors: A national survey in China. PLoS One, 11(11), Article e0165341. https://doi.org/10.1371/journal.pone.0165341

Zeng, Y. Y., Wu, M. X., Geng, D. D., Cheng, L., Zhou, S. N., Fan, K. L., Yu, X., Tang, W. J., & He, J. C. (2021). Early-onset depression in stroke patients: effects on unfavorable outcome 5 years post-stroke. Frontiers in Psychiatry, 12, Article 556981. https://doi.org/10.3389/fpsyt.2021.556981
