# Supplementary material for: Association between post-stroke depression and functional outcomes: A systematic review
Source: PLoS One. 2024 Aug 22;19(8):e0309158. doi: 10.1371/journal.pone.0309158 (PMC11341015; doi:10.1371/journal.pone.0309158)
Supplement: S3 Table — (DOCX) [file pone.0309158.s003.docx]

**S3 Table. The methodological quality of the included studies.**

| **JBI Critical Appraisal Checklist for cohort studies** | | | | | |  |  |  |  |  |  |  |  |  |  |  |
| --- | --- | --- | --- | --- | --- | --- | --- | --- | --- | --- | --- | --- | --- | --- | --- | --- |
| **Appraisal questions/References** | Ayerbe et al., 2015 | El Husseini et al., 2017 | Kang et al., 2018 | Kapoor et al., 2019 | Karaahmet et al., 2017 | Kim et al., 2022 | Li et al., 2019 | Lopatkiewicz et al., 2021 | Lv et al., 2021 | Matsuzaki et al., 2015 | Schöttke et al., 2020 | Sharma et al., 2021 | Wang et al., 2018 | Yang et al., 2016 | Zeng et al., 2021 | Nakamori et al., 2020 |
| 1. Were the two groups similar and recruited from the same population? | + | + | + | + | + | + | ^ | + | + | + | + | + | + | + | + | + |
| 2. Were the exposures measured similarly to assign people to both exposed and unexposed groups? | + | + | + | + | + | + | + | + | + | + | + | + | + | + | + | + |
| 3. Was the exposure measured in a valid and reliable way? | + | + | + | + | + | + | + | + | + | + | + | + | + | + | + | + |
| 4. Were confounding factors identified? | + | + | + | + | + | + | + | + | + | + | + | - | + | + | + | + |
| 5. Were strategies to deal with confounding factors stated? | + | + | + | + | - | + | + | + | + | + | + | - | + | + | + | + |
| 6. Were the groups/participants free of the outcome at the start of the study (or at the moment of exposure)? | - | - | - | - | - | - | - | - | - | - | - | - | - | - | - | - |
| 7. Were the outcomes measured in a valid and reliable way? | + | + | + | + | + | + | + | + | + | + | + | + | + | + | + | + |
| 8. Was the follow up time reported and sufficient to be long enough for outcomes to occur? | + | + | + | + | + | ? | + | + | + | + | + | + | + | + | + | + |
| 9. Was follow up complete, and if not, were the reasons to loss to follow up described and explored? | + | + | + | + | + | ? | + | + | + | + | + | + | + | + | + | + |
| 10. Were strategies to address incomplete follow up utilized? | + | + | + | + | + | ? | + | + | + | + | + | + | + | + | + | + |
| 11. Was appropriate statistical analysis used? | + | + | + | + | + | + | + | + | + | + | + | + | + | + | + | + |
| ***Percentage of yes* (%)*** | 90.9 | 90.9 | 90.9 | 90.9 | 81.8 | 63.6 | 81.8 | 90.9 | 90.9 | 90.9 | 90.9 | 72.7 | 90.9 | 90.9 | 90.9 | 90.9 |
| **JBI Critical Appraisal Checklist for analytical cross-sectional studies** | | | | | |  |  |  |  |  |  |  |  |  |  |  |
| **Appraisal questions/References** | Blomgren et al., 2019 | Ezema et al., 2019 | Ghaffari et al., 2021 | Mohammed et al., 2023 |  |  |  |  |  |  |  |  |  |  |  |  |
| 1. Were the criteria for inclusion in the sample clearly defined? | + | + | + | + |  |  |  |  |  |  |  |  |  |  |  |  |
| 2. Were the study subjects and the setting described in detail? | + | + | + | + |  |  |  |  |  |  |  |  |  |  |  |  |
| 3. Was the exposure measured in a valid and reliable way? | + | + | + | + |  |  |  |  |  |  |  |  |  |  |  |  |
| 4. Were objective, standard criteria used for measurement of the condition? | + | + | + | + |  |  |  |  |  |  |  |  |  |  |  |  |
| 5. Were confounding factors identified? | + | + | + | + |  |  |  |  |  |  |  |  |  |  |  |  |
| 6. Were strategies to deal with confounding factors stated? | + | + | + | + |  |  |  |  |  |  |  |  |  |  |  |  |
| 7. Were the outcomes measured in a valid and reliable way? | + | + | + | + |  |  |  |  |  |  |  |  |  |  |  |  |
| 8. Was appropriate statistical analysis used? | + | + | + | + |  |  |  |  |  |  |  |  |  |  |  |  |
| ***Percentage of yes* (%)*** | 100.0 | 100.0 | 100.0 | 100.0 |  |  |  |  |  |  |  |  |  |  |  |  |
| **JBI Critical Appraisal Checklist for case control studies** | | | | | |  |  |  |  |  |  |  |  |  |  |  |
| **Appraisal questions/References** | Paolucci et al., 2019 |  |  |  |  |  |  |  |  |  |  |  |  |  |  |  |
| 1. Were the groups comparable other than the presence of disease in cases or the absence of disease in controls? | + |  |  |  |  |  |  |  |  |  |  |  |  |  |  |  |
| 2. Were cases and controls matched appropriately? | + |  |  |  |  |  |  |  |  |  |  |  |  |  |  |  |
| 3. Were the same criteria used for identification of cases and controls? | + |  |  |  |  |  |  |  |  |  |  |  |  |  |  |  |
| 4. Was exposure measured in a standard, valid and reliable way? | + |  |  |  |  |  |  |  |  |  |  |  |  |  |  |  |
| 5. Was exposure measured in the same way for cases and controls? | + |  |  |  |  |  |  |  |  |  |  |  |  |  |  |  |
| 6. Were confounding factors identified? | - |  |  |  |  |  |  |  |  |  |  |  |  |  |  |  |
| 7. Were strategies to deal with confounding factors stated? | - |  |  |  |  |  |  |  |  |  |  |  |  |  |  |  |
| 8. Were outcomes assessed in a standard, valid and reliable way for cases and controls? | + |  |  |  |  |  |  |  |  |  |  |  |  |  |  |  |
| 9. Was the exposure period of interest long enough to be meaningful? | + |  |  |  |  |  |  |  |  |  |  |  |  |  |  |  |
| 10. Was appropriate statistical analysis used? | + |  |  |  |  |  |  |  |  |  |  |  |  |  |  |  |
| ***Percentage of yes* (%)*** | 80.0 |  |  |  |  |  |  |  |  |  |  |  |  |  |  |  |
| "+": yes; "-": no; "?": unclear; "^": not applicable | | | | | |  |  |  |  |  |  |  |  |  |  |  |
| *The number of "yes"/ total items in the checklist x 100. | | | | | |  |  |  |  |  |  |  |  |  |  |  |
