# Supplementary material for: Association between post-stroke depression and functional outcomes: A systematic review
Source: PLoS One. 2024 Aug 22;19(8):e0309158. doi: 10.1371/journal.pone.0309158 (PMC11341015; doi:10.1371/journal.pone.0309158)
Supplement: Attachment — Submitted filename: Responses to reviwer comments-R1-PONE-D-24-26302.docx [file pone.0309158.s006.docx]

Response letter to the reviewer(s)' comments on manuscript ID PONE-D-24-26302

**Title:** “Association between post-stroke depression and functional outcomes: A systematic review”

Dear Editor,

Thank you for considering our manuscript, “Association between post-stroke depression and functional outcomes: A systematic review.” All authors are very grateful for all the constructive and valuable comments from the two reviewers, which have helped us improve this revised version of the manuscript. Please kindly express our appreciation to each one of them. We would like to provide the point-by-point responses described in the table to the reviewers’ comments as well as journal requirements. Please kindly find the detailed responses to reviewers’ comments in the following orders: response to Journal requirements on pages 1-2, Reviewer 1 on pages 2-5, and Reviewer 2 on page 5.

| **Journal Requirements:** | **Authors’ Responses** |
| --- | --- |
| 1. Please ensure that your manuscript meets PLOS ONE's style requirements, including those for file naming. The PLOS ONE style templates can be found at  <https://journals.plos.org/plosone/s/file?id=wjVg/PLOSOne_formatting_sample_main_body.pdf> and  <https://journals.plos.org/plosone/s/file?id=ba62/PLOSOne_formatting_sample_title_authors_affiliations.pdf> | We checked PLOS ONE's style templates for formatting guidelines for manuscript body, title, author, and affiliation. We ensure that the manuscript meets PLOS ONE's style requirements. |
| 2. When completing the data availability statement of the submission form, you indicated that you will make your data available on acceptance. We strongly recommend all authors decide on a data sharing plan before acceptance, as the process can be lengthy and hold up publication timelines. Please note that, though access restrictions are acceptable now, your entire data will need to be made freely accessible if your manuscript is accepted for publication. This policy applies to all data except where public deposition would breach compliance with the protocol approved by your research ethics board. If you are unable to adhere to our open data policy, please kindly revise your statement to explain your reasoning and we will seek the editor's input on an exemption. Please be assured that, once you have provided your new statement, the assessment of your exemption will not hold up the peer review process. | All authors have decided on the data sharing plan before acceptance. The data availability statement is “All relevant data are within the manuscript and its Supporting Information files.” A change in the system would be made to the resubmission process. |
| 3. Please review your reference list to ensure that it is complete and correct. If you have cited papers that have been retracted, please include the rationale for doing so in the manuscript text, or remove these references and replace them with relevant current references. Any changes to the reference list should be mentioned in the rebuttal letter that accompanies your revised manuscript. If you need to cite a retracted article, indicate the article’s retracted status in the References list and also include a citation and full reference for the retraction notice. | We checked our 53 references on the “Retraction Watch Databases” website and found no retraction paper cited in this recent manuscript. |

| **Reviewer 1’s Comments** | **Authors’ Responses** |
| --- | --- |
| 1. An assessment of the risk of bias in observational epidemiological studies in the original studies was omitted. Review authors should categorize risk of bias as low, medium, or high. It would have been important for the review authors to assess the risk of bias among the included studies. | 1) Thank you for your suggestion.  In this systematic review, we used Joanna Briggs Institute (JBI) critical appraisal tools for analytical cohort, cross-sectional, and case-control studies to assess the risk of bias among included studies as mentioned in the Methods and Results sections, and details of the assessment are provided in supplementary material S3 Table. Moreover, we added more explanation of methodological quality as follows:  In Methods section  - Assessment of methodological quality (page 7; line 151).  - Studies with a JBI score higher than 70% were categorized as high quality, moderate quality if scored between 50% and 70%, and low quality if scored lower than 50% (page 7; lines 157-159).  In Results section  - Methodological quality assessment of the included studies which explained that twenty (95.2%) studies were classified as high quality (low risk of bias), one (4.8%) study was classified as moderate quality (unclear risk of bias). No study with low quality (high risk of bias) was included in this review (pages 11-12; lines 222, 227-229).  Moreover, the assessment of methodological quality was also added in Table 4 (pages 19-21). |
| 2. No assessment of publication bias among the included studies is presented in the manuscript. It would have been important for the review authors to at least create a scatter (i.e., funnel) plot of the studies plotting precision (standard error) or sample size by the effect size (mean difference) to assess for publication bias. | 1) Thank you for your suggestion.  Publication bias in the form of a scatter plot could not be provided for this review due to significant variations in study design, timing of assessment, depression assessment tools, functional outcome measures, and form of reporting depression and functional outcomes among included papers. Moreover, PSD was the factor that could not be assigned to participants, so it was difficult to identify the case and control group. Although some studies reported sample size and means of PSD and non-PSD participants, they used different tools in measuring functional outcomes, and only two papers reported the same things in that they used the same assessment measures and times. These did not allow us to create a scatter plot of the studies for publication bias assessment. Therefore, we added more explanation and discussion on these limitations in the Methods and Results sections.  The Methods section (page 8; lines 172-173) explained that data synthesis was done narratively since there was heterogeneity in included study characteristics.  In the Results section, details are shown as follows: **- Degree of dependence using mRS** (page 15; line 278-280) explained that all included studies that assessed mRS reported significant variation in terms of timing of assessment (discharge, 3 months, 12 months, and five years), depression assessment tools (6 different tools), form of report degree of dependence (means or binary grouping). Therefore, these results are presented narratively.  **- Basic ADL** (page 16; line 301-303) added that included studies assessed ADL reported significant variation in terms of study designs (cohort, cross-sectional, and case-control), timing of assessment (discharge, 6 weeks, 3 months, 12 months, 2 years, 3 years, five years, and not specific time point), depression assessment tools (7 different tools), form of report ADL (means or groups). Therefore, studies within this group are reported narratively.  **- IADL** (pages 16-17; line 322-324) explained that included studies assessed IADL reported significant variation in terms of study designs (cohort, cross-sectional), timing of assessment (3 months, 2 years, seven years, and unidentified), depression assessment tools (5 different tools), form of report IADL (means or groups). Therefore, these results are presented narratively.  **- Physical and cognitive function** (page 17; line 342-344), there were only three studies that utilized FIM measurement to measure this functional outcome and these three papers had significant variation in terms of study designs (cohort, cross-sectional), timing of assessment (3 months, 2 years, seven years, and unidentified), depression measures (HADS-D, BDI, PHQ-2, CES-D), reporting of physical and cognitive function. Studies within this group were therefore narratively presented. |
| 3. Another concern is a lot of work by the review authors went into performing this systematic review in an attempt to answer the important question about whether or not post-stroke depression is truly associated with functional outcomes. Unfortunately, the blend of different observational study designs most likely led to significant heterogeneity among the studies, due to variability rather than from random chance alone. Based on the finding of this review, please clarify how the review authors concluded that post-stroke depression is significantly associated with poor functional outcomes, rather than attributing these outcomes directly to the stroke itself? Additionally, to what extent does comorbidity influence patients’ functional outcomes? | 1) Thank you for your suggestion.  We have discussed more about review results and made conclusions based on review findings and limitations as suggested (page 26; lines 455-471). |

| **Reviewer 2’s Comments** | **Authors’ Responses** |
| --- | --- |
| Please elaborate in the discussion the importance of the findings, and the way forward. In the limitation, please discuss the different research designs in the selected papers, and how this may affect the prevalence and factors associated. | 1) Thank you for your suggestion.  The importance of the findings was elaborated in the Discussion section (page 26; lines 455-471). Moreover, the limitations of selected papers were discussed and explained more as suggested. (page 26; lines 455-471, page 27; lines 477-479). |
